# Cytotoxicity of the Sesquiterpene Lactone Spiciformin and Its Acetyl Derivative against the Human Leukemia Cell Lines U-937 and HL-60

**DOI:** 10.3390/ijms21082782

**Published:** 2020-04-16

**Authors:** Ester Saavedra, Francisco Estévez-Sarmiento, Mercedes Said, José Luis Eiroa, Sara Rubio, José Quintana, Francisco Estévez

**Affiliations:** 1 Departamento de Bioquímica y Biología Molecular, Instituto Universitario de Investigaciones Biomédicas y Sanitarias (IUIBS), Universidad de Las Palmas de Gran Canaria, 35016 Las Palmas de Gran Canaria, Spain; saavedradiazestergloria@gmail.com (E.S.); festevez1985@gmail.com (F.E.-S.); mercedessaid@gmail.com (M.S.); sara.rubio@ulpgc.es (S.R.); jose.quintana@ulpgc.es (J.Q.); 2 Departamento de Química, Instituto Universitario de Investigaciones Biomédicas y Sanitarias (IUIBS), Universidad de Las Palmas de Gran Canaria, 35017 Las Palmas de Gran Canaria, Spain; joseluis.eiroa@ulpgc.es

**Keywords:** apoptosis, cytotoxicity, caspase, poly(ADP-ribose) polymerase, sesquiterpene lactone

## Abstract

Spiciformin (**1**) is a sesquiterpene lactone with a germacrane skeleton that is found in two *Tanacetum* species endemic to the Canary Islands. In this study, the cytotoxicities of **1** and its acetyl derivative (**2**) were evaluated against human tumor cells. These sesquiterpene lactones were cytotoxic against human acute myeloid leukemia (U-937 and HL-60) cells, even in cells over-expressing the pro-survival protein Bcl-2, but melanoma (SK-MEL-1) and human mononuclear cells isolated from blood of healthy donors were more resistant. Both compounds are apoptotic inducers in human leukemia U-937 cells. Cell death was mediated by the processing and activation of initiator and effector caspases and the cleavage of poly(ADP-ribose) polymerase, and it was blocked by a broad-spectrum caspase inhibitor and (in the case of sesquiterpene lactone **2**) by the selective caspase-3/7, -8, and -9 inhibitors. In addition, certainly in the case of compound **2**, this was found to be associated with a decrease in mitochondrial membrane potential, downregulation of the anti-apoptotic protein Bcl-2, activation of the mitogen-activated protein kinases signaling pathway, and generation of reactive oxygen species. It will, therefore, be relevant to continue characterization of this class of compounds.

## 1. Introduction

The discovery of new anticancer agents is of great interest since the currently available drugs against cancer exhibit several critical problems, including serious adverse effects, insufficient effectiveness, and the development of multidrug resistance [1]. Acute myeloid leukemia is a lethal form of hematologic malignancy and is the most usual type of acute leukemia in adults and accounts for 15%-20% of all tumors diagnosed in children aged 1 to 14 years [2]. Despite recent advances to treat and cure acute myeloid leukemia, mortality rates are still very high [3].

Many small molecules approved as anticancer drugs are based on natural products [4]. Sesquiterpene lactones are naturally occurring compounds formed from the condensation of three isoprene units and contain one or more lactone rings. A large number of sesquiterpene lactones exhibit cancer cell cytotoxicity, which depends on the presence of the α-methylene-γ-lactone moiety. This functional group acts as an alkylating agent in a Michael-type reaction with biomolecules containing nucleophilic groups [5,6]. Sesquiterpene lactones target multiple signal transduction pathways involved in survival and cell death. It has been shown that sesquiterpene lactones inhibit nuclear factor κB, phosphatidylinositol-3-kinase, Janus kinase/signal transducer, and activator of transcription and mitogen-activated protein kinase signaling, as well as epigenetic factors such as DNA methyltransferase 1 and histone deacetylase 1, together with activation of c-Jun *N*-terminal kinases and reactive oxygen species generation (reviewed in Reference [6]). The effect of sesquiterpene lactones on multiple targets triggers the blocking of cancer development and progression and might sensitize cancer cells to conventional chemotherapy. Interest in this kind of natural product has emerged mainly because they are selective toward tumor and cancer stem cells [7].

Several sesquiterpene lactones have been evaluated in cancer clinical trials [8]. Specific sesquiterpene lactones are apoptotic inducers in different types of cancer cells [9]. This type of regulated cell death is characterized by cytoplasmic shrinkage, plasma membrane blebbing, phosphatidylserine exposure at the cell surface, loss of mitochondrial membrane potential, chromatin condensation, nuclear fragmentation, and formation of apoptotic vesicles [10,11]. It can occur with or without the activation of a class of aspartate-specific cysteine proteases known as caspases [12,13,14]. Two main apoptotic pathways have been described, namely the extrinsic and intrinsic pathways [15]. The intrinsic or mitochondrial pathway is controlled by pro-apoptotic and anti-apoptotic proteins of the B-cell lymphoma 2 (Bcl-2) family and involves mitochondrial outer membrane permeabilization, which promotes the cytosolic release of apoptogenic factors including cytochrome *c* [16]. Cytosolic cytochrome *c* is involved in the assembly of apoptosome and pro-caspase-9 stimulation, which activates the executioner caspase-3 and -7. The death receptor or extrinsic pathway is mediated by plasma membrane receptors and induces activation of caspase-8, which mainly activates caspase-3 [17,18]. In addition, most of the anticancer drugs trigger apoptosis induction in malignant cells and reactivation of this pathway is critical for more effective treatments [19].

**Figure 1:**
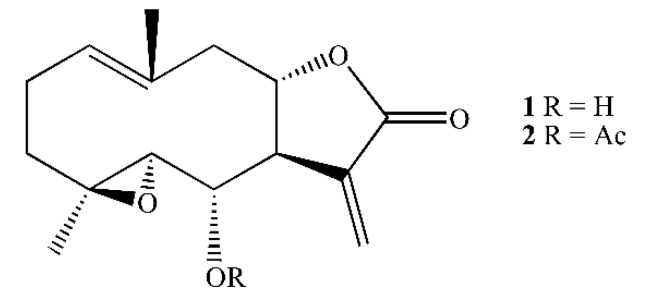
Structure of spiciformin (**1**) and its acetate (**2**).

Two *Tanacetum* species endemic of the Canary Islands, *Tanacetum ptarmiciflorum* and *T. ferulaceum* var. *latipinnum* contain spiciformin (**1**) (Figure 1), a sesquiterpene lactone with a germacrane skeleton [20]. Its potential antiproliferative activity against human cancer cells has not been investigated to date. The justification for studying the cytotoxic effects of spiciformin against human cancer cells is two-fold: (1) it has the same skeleton as parthenolide, a sesquiterpene lactone with potent anti-cancer and anti-inflammatory activity, and (2) its chemical structure allows the addition of new substituents to improve its cytotoxicity. The present study explores the potential cytotoxicity of spiciformin (**1**) and its acetyl derivative (**2**) against human tumor cells and their underlying mechanisms of cell death, including the disruption of mitochondrial membrane potential, the activation of the caspase cascade and the mitogen-activated protein kinase pathway, the changes in the Bcl-2 family proteins expression, and the generation of reactive oxygen species.

## 2. Results and Discussion

### 2.1. Spiciformin Acetate (**2**) is More Cytotoxic Than Spiciformin (**1**) Against Human Tumor Cells

In the present study, the effects of the naturally occurring spiciformin (**1**), an epoxylated germacranolide, and its acetyl derivative (**2**) on the growth of human acute myeloid leukemia (U-937 and HL-60) and melanoma (SK-MEL-1) cells were evaluated. Spiciformin acetate (**2**) was synthesized and evaluated for cytotoxicity against human tumor cells since its higher hydrophobicity can facilitate diffusion through the plasma membrane and, therefore, enhance cytotoxicity in vitro. No studies have yet addressed the cytotoxicity of these germacranolides against human acute myeloid leukemia and melanoma cell lines.

**Figure 2:**
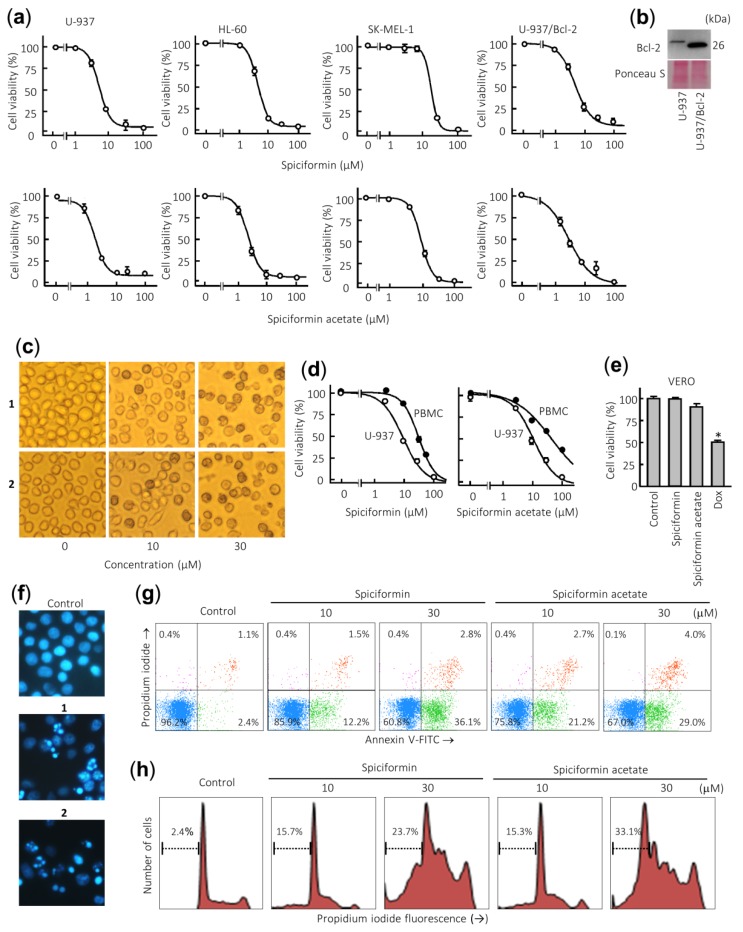
Effects of sesquiterpene lactones **1** and **2** on human tumor cells. (**a**) Dose–response study on human cancer cell lines. Cells were cultured in the presence of increasing concentrations of spiciformin or spiciformin acetate for 72 h, and then the cell viability was determined by the 3-(4,5-dimethyl-2-thiazolyl)-2,5-diphenyl-2*H*-tetrazolium bromide (MTT) assay. (**b**) Immunoblot analysis of Bcl-2 protein in U-937 and U-937/Bcl-2. The membrane was stained with Ponceau S to control equal protein loading. (**c**) U-937 cells were incubated with the indicated concentrations of the specified compounds for 24 h and visualized with an inverted phase-contrast microscope. Original magnification 40×. (**d**) Differential effects of sesquiterpene lactones on viability of normal mononuclear cells from peripheral blood (PBMCs) versus U-937 cells. Quiescent PBMC from healthy human origin and human leukemia cells were treated with increasing concentrations of **1** and **2** for 24 h. (**e**) Effect of 100 µM sesquiterpene lactones on Vero cells viability determined by the MTT assay after treatment for 24 h. Doxorubicin (Dox, 3 µM) was included as a positive control. The results are means ± SE of three independent experiments performed in triplicate (* *p* < 0.05, significantly different from untreated control). (**f**) Morphological analysis after staining with Hoechst 33,258 to assess nuclear chromatin fragmentation and condensation (i.e., apoptosis) after treatment with 30 µM sesquiterpene lactones **1** and **2**. Cells were observed under a fluorescence microscope and representative fields were photographed with a digital camera. Original magnification 63×. (**g**) Cells were incubated with the specified concentrations of germacranolides **1** and **2** for 24 h, stained with annexin V-FITC and propidium iodide and analyzed by flow cytometry. Shown data are representative out of three independent experiments with similar results. (**h**) Cells were incubated as in (**e**), fixed, and analyzed by flow cytometry. Representative histograms of flow cytometry after propidium iodide labeling and the percentages of hypodiploid cells (apoptotic cells) are shown.

To evaluate the potential antiproliferative effect of both sesquiterpene lactones, cells were treated with increasing concentrations and viability was determined by the 3-(4,5-dimethyl-2-thiazolyl)-2,5-diphenyl-2*H*-tetrazolium bromide (MTT) assay. These compounds were found to inhibit the cell viability in a concentration-dependent manner and displayed cytotoxic effects against the human leukemia cell lines (Figure 2a). The IC_50_ (concentrations inducing a 50% inhibition of cell growth) values for spiciformin (**1**) were similar in both leukemia cells (IC_50_ values of ca. 5 µM), while in SK-MEL-1 the IC_50_ value was approximately four-fold higher (Table 1). The human tumor U-937 cells were more sensitive than SK-MEL-1 melanoma cells to the cytotoxic effects of spiciformin (**1**). The IC_50_ values for spiciformin acetate (**2**) were lower than spiciformin (**1**) in all cell lines assayed, except in HL-60 cells. In these experiments, etoposide, which is an agent commonly used for the treatment of acute leukemia, was included as a positive control. The IC_50_ values of etoposide were 1.2 ± 0.4 µM, 0.4 ± 0.1 µM, and 10 ± 4 µM, for U-937, HL-60, and SK-MEL-1, respectively.

Over-expression of the pro-survival protein Bcl-2 did not suppress the cytotoxicity induced by sesquiterpene lactones **1** and **2** since the IC_50_ values were similar for U-937 and U-937/Bcl-2 cells (Figure 2b, Table 1). This result is very interesting since Bcl-2 protein confers resistance to apoptosis by antagonizing the mitochondrial outer membrane permeability and exerts anti-apoptotic functions through the binding of pro-apoptotic members of the Bcl-2 family [21].

Treatment with both sesquiterpene lactones induced profound morphological changes as well as a marked reduction in the number of cells. Representative images obtained with an inverted phase-contrast microscope of U-937 cells treated with increasing concentrations of spiciformin (**1**) and spiciformin acetate (**2**) for 24 h are shown in Figure 2c. It was also investigated whether both sesquiterpene lactones were likewise cytotoxic for human mononuclear cells isolated from peripheral blood of healthy donors (PBMC). As shown in Figure 2d, a pronounced reduction was detected in the proliferation of U-937 cells while quiescent PBMC showed higher resistance than leukemia cells, even at 30 µM sesquiterpene lactones. The IC_50_ values determined at 24 h for **1** and **2** were 30.4 ± 3.6 µM and 41.0 ± 3.8 µM against PBMC, while the IC_50_ values against U-937 cells were 10 ± 4.0 µM and 12.2 ± 2.6 µM, respectively. Interestingly, the IC_50_ values in U-937 cells for both compounds were similar at 24 h. These results suggest that both sesquiterpene lactones affected viability of peripheral blood mononuclear cells (PBMC) to a lesser extent than U-937 cells. Control experiments with the fibroblast-like Vero cell line showed no appreciable toxicity even at 100 µM spiciformin or spiciformin acetate for 24 h. As a positive control, doxorubicin (3 µM) was included in the experiment and there was a 50% decrease in the viability of these cells (Figure 2e). Future studies addressing the selectivity and efficacy of in vivo concentrations of these compounds are necessary to determine their potential for human health.

### 2.2. Spiciformin (**1**) and Spiciformin Acetate (**2**) Induce Apoptosis in Human Acute Myeloid Leukemia Cells

To elucidate whether growth inhibition mediated by sesquiterpene lactones was caused by apoptosis, fluorescence microscopy using Hoechst 33258 staining and flow cytometry experiments were performed in U-937 cells. To this end, cells were treated with 30 µM of compounds for 24 h, and morphological aspects were visualized by fluorescent microscopy. As shown in Figure 2f, control cells showed round nuclei with uncondensed and dispersed chromatin, while treated cells with sesquiterpene lactones showed fragmented and condensed nuclei. The evaluation of the number of annexin V-fluoresceine isothiocyanate (FITC) positive cells by flow cytometry revealed that the percentage of apoptosis increased about five and nine times in cells treated with 10 µM sesquiterpene lactones **1** and **2** for 24 h, respectively (Figure 2g). In accordance with the annexin V-FITC studies, the percentage of hypodiploid cells (i.e., sub-G_1_ fraction) increased about 10-fold and 14-fold in cells treated with compounds **1** and **2**, respectively, compared with control cells after 24 h exposure at a concentration of 30 µM. Representative histograms obtained after treatment with both sesquiterpene lactones and subjected to flow cytometric analysis of nuclei stained with propidium iodide are shown (Figure 2h). These results indicate that sesquiterpene lactones **1** and **2** induce apoptosis in human U-937 cells.

### 2.3. Spiciformin (**1**) and Spiciformin Acetate (**2**) Induce Cell Death by a Caspase-Dependent Pathway 

**Figure 3:**
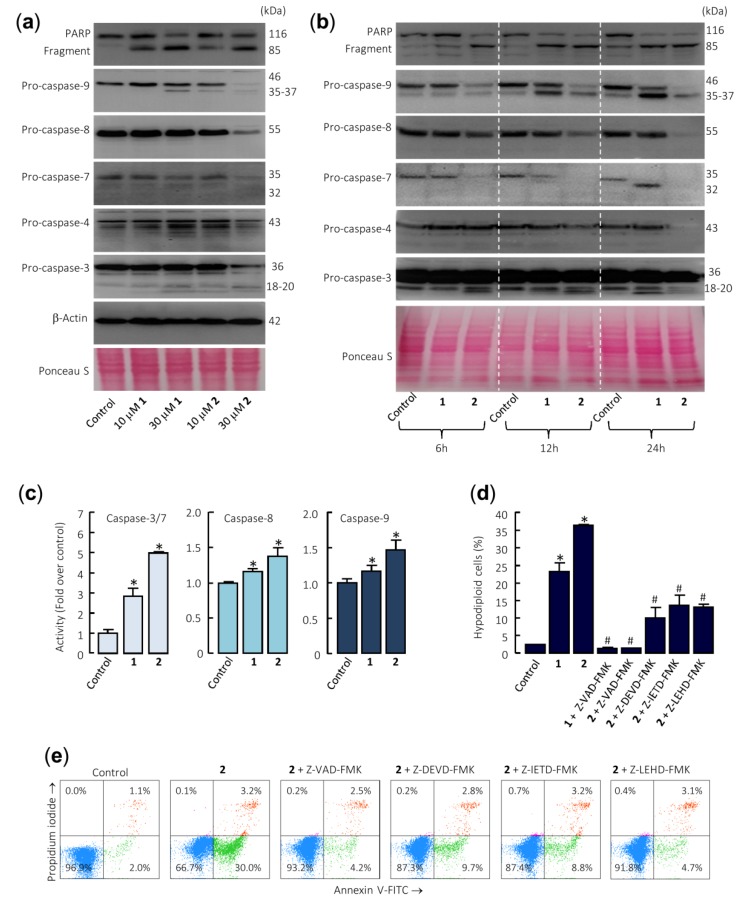
Caspase cascade is involved in apoptosis induction by sesquiterpene lactones in U-937 cells. (**a**) The cells were cultured for 24 h in control conditions or in the presence of the indicated concentrations of the specified compounds, and the cell lysates were analyzed by Western blots for the cleavage of poly(ADP-ribose) polymerase (PARP) and pro-caspases-3, -4, -7, -8, and -9. β-Actin was used as a loading control. Total protein loading was also controlled by Ponceau S staining (a representative stained membrane is shown). (**b**) Representative Western blots show the time-dependent PARP cleavage and pro-caspases processing in response to sesquiterpene lactones 1 and 2. Cells were incubated with 30 µM of compounds for the indicated time points, and protein extracts were prepared and analyzed by immunoblotting using specific antibodies. The membrane was stained with Ponceau S to control equal protein loading. (**c**) Cells were cultured in control conditions or in the presence of 30 µM sesquiterpene lactones for 24 h and lysates were assayed for caspase-3/7, -8, and -9 activities. Results are expressed as fold increase in caspase activity compared with control. Values represent means ± SE of three independent experiments, each performed in triplicate (* *p* < 0.05, significantly different from untreated control). (**d**) Effects of caspase inhibitors on the percentage of hypodiploid cells. The cells were pretreated in the absence or presence of Z-VAD-FMK (100 µM) or the selective caspase inhibitors Z-DEVD-FMK (50 µM), Z-IETD-FMK (50 µM), or Z-LEHD-FMK (50 µM) for 1 h before the addition of sesquiterpene lactones (30 µM). The percentages of hypodiploid cells were determined by flow cytometry. The data represent means ± SE of two independent experiments with three determinations each (* *p* < 0.05, significantly different from untreated control, # *p* < 0.05, significantly different from sesquiterpene lactone treatment alone). (**e**) Cells were pretreated with the indicated caspase inhibitors and then with 30 µM compound **2**, harvested, stained with annexin V-FITC and propidium iodide, and analyzed by flow cytometry.

To determine whether compounds **1** and **2** activate the caspase cascade, U-937 cells were incubated in the absence or presence of both sesquiterpene lactones and poly(ADP-ribose) polymerase (PARP), as well as the initiator (caspase-8 and -9) and executioner (caspase-3 and -7) caspases were determined by immunoblotting using specific antibodies. Dose–response and time–course experiments revealed that both compounds induce PARP cleavage and the processing of pro-caspases-3, -7, and -9 (Figure 3a,b). The processing of pro-caspase-8 (detected as a reduction of this zymogen) was evident even with the lower concentration of compound **2** (10 µM) and PARP cleavage was detected at concentrations as low as 10 µM of each sesquiterpene lactone (Figure 3a). Caspase-4, which is involved in the endoplasmic reticulum stress, was also cleaved in U-937 cells treated with **1** and **2**. In these experiments, Ponceau S staining prior to antibody detection was used as an alternative loading control.

Enzymatic assays were also performed to confirm caspase activation. To this end, U-937 cells were incubated in the presence of 30 µM sesquiterpene lactones **1** and **2** for 24 h and cell lysates were analyzed for cleavage of the tetrapeptide substrates DEVD-*p*NA, IETD-*p*NA, and LEHD-*p*NA as specific substrates of caspase-3/7, -8 and -9, respectively. The results revealed that both sesquiterpene lactones stimulate the activity of the initiator caspases, caspase-8 and -9, as well as the effector caspases-3/7. In addition, compound **2** was a more potent caspase activator than compound **1** (Figure 3c). To determine whether caspases are involved in sesquiterpene lactones-induced cell death, it was investigated the effects of the pancaspase inhibitor Z-VAD-FMK, as well as the following selective caspase inhibitors: the caspase-3/7 inhibitor Z-DEVD-FMK, the caspase-4 inhibitor Ac-LEVD-CHO, the caspase-8 inhibitor Z-IETD-FMK, and the caspase-9 inhibitor Z-LEHD-FMK. Pretreatment of cells with Z-VAD-FMK completely blocked the increase in the percentage of sub-G_1_ cells (Figure 3d) as well as the increase in the percentage of annexin V-FITC positive cells (Figure 3e) induced by compounds **1** and **2**. In addition, the selective caspase inhibitors Z-DEVD-FMK, Z-IETD-FMK, and Z-LEHD-FMK significantly reduced the percentage of sub-G_1_ cells and almost completely stopped annexin V-FITC positive cells from being induced by compound **2** (Figure 3d,e). Nonetheless, the pretreatment of cells with the selective caspase-4 inhibitor Ac-LEVD-CHO did not reduce the percentage of annexin V-FITC positive cells induced by compound **2** (results not shown).

Taken together, these results demonstrate that both sesquiterpene lactones induce apoptosis by a mechanism dependent on caspase since cell death was completely inhibited by the broad-spectrum caspase inhibitor Z-VAD-FMK. In addition, the selective caspase inhibitors for caspase-3 and -7, -8, and -9 were effective at blocking the increase in the percentage of annexin V positive cells and the increase in the percentage of hypodiploid cells induced by these sesquiterpene lactones. This result suggests that the mechanism of cytotoxicity is dependent on caspase-8 and caspase-9 activation. Although pro-caspase-4 was also processed by these sesquiterpene lactones, the selective caspase-4 inhibitor did not block cell death, suggesting a minor role of the endoplasmic reticulum stress signaling pathway in the mechanism of cell death.

The mechanism of cytotoxicity exhibited by spiciformin (**1**) and spiciformin acetate (**2**) is different from the previously described sesquiterpene lactones depending on the cell types. For example, parthenolide, a sesquiterpene lactone isolated from the plant *Tanacetum parthenium*, induces cell death that is not dependent on caspases in human osteosarcoma MG63 and melanoma SK-MEL-28, because cell death was not blocked by the pan-caspase inhibitor Z-VAD-FMK [22]. Parthenolide cytotoxicity has also been described to be partly caspase-dependent, as the broad-spectrum caspase inhibitor Z-VAD-FMK could partially protect multiple myeloma cells [23].

**Figure 4:**
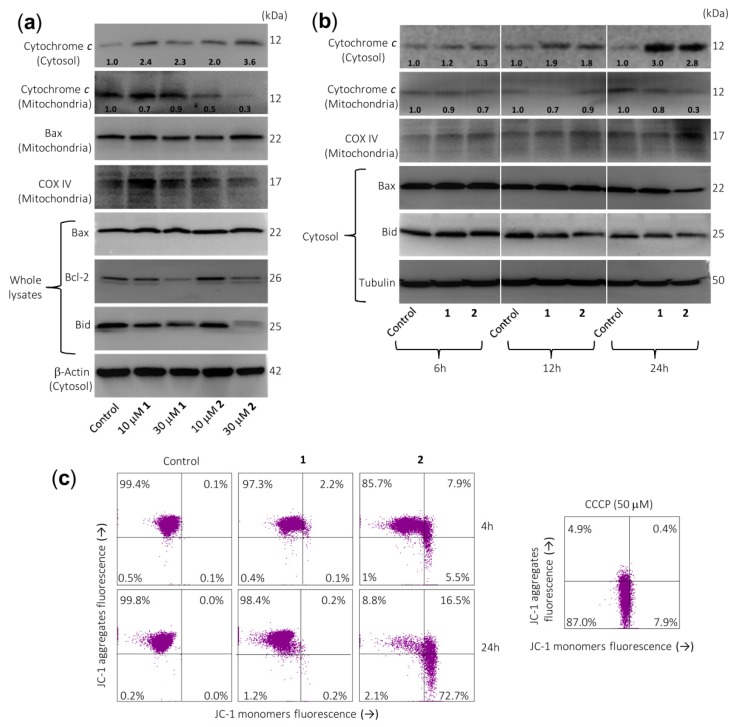
Sesquiterpene lactones induce the release of cytochrome *c* and changes on Bcl-2 family members’ expression in U-937 cells. (**a**) Cells were cultured in control conditions or in the presence of the indicated concentrations of sesquiterpene lactones for 24 h and cytosolic and mitochondrial extracts were analyzed by immunoblotting to detect mitochondrial cytochrome *c* release and Bcl-2 family proteins, except for Bcl-2 and Bax, which were analyzed in whole extracts. Bax was also analyzed in the mitochondrial fraction. β-Actin and COX IV (cytochrome *c* oxidase) were used as loading controls in cytosol and mitochondria, respectively. (**b**) Cells were incubated in control conditions or in the presence of 30 µM compounds **1** or **2** for the indicated times and cytosolic or mitochondrial fractions were prepared and analyzed by immunoblotting using specific antibodies described in the Material and Methods section. Tubulin and COX IV (cytochrome *c* oxidase) were used as loading controls in cytosol and mitochondria, respectively. The cytosolic and mitochondria-enriched fractions were prepared and Western blot analyses were performed as described in the Materials and Methods. Numbers below each panel of cytochrome *c* indicate fold differences after normalization to the corresponding loading control (β-actin, tubulin, or COX IV). (**c**) Sesquiterpene lactone **2** reduces the mitochondrial membrane potential (ΔΨm). Cells were incubated in control conditions or in the presence of 30 µM compounds **1** or **2** for 4 h or 24 h, and ΔΨm were analyzed by flow cytometry using the fluorescent probe JC-1. The results are representative of three separate experiments. Carbonyl cyanide *m*-chlorophenylhydrazone (CCCP, 50 µM) was used as a positive control.

Since the intrinsic pathway of apoptosis involves release of mitochondrial proteins such as cytochrome *c* into the cytosol, it was explored whether this crucial protein is involved in the cell death induced by both sesquiterpene lactones. Dose–response experiments revealed an increase in cytochrome *c* in the cytosol after 24 h of treatment with 10 µM of compound **2** (Figure 4a). This was accompanied by a decrease in cytochrome *c* in the mitochondria-enriched fraction. However, results at earlier time points (6 h or 12 h; Figure 4b) remain largely elusive for both compounds. Control determinations using anti-cytochrome *c* oxidase (COX IV) antibody revealed that the supernatants of the cytosolic fraction were free of mitochondrial contamination. Therefore, sesquiterpene lactones have reasonably been shown to induce late release of cytochrome *c* as well as activation of multiple caspases, including caspase-3, -4, -7, -8, and -9, emphasizing that both the extrinsic and the intrinsic pathways play a role in the observed cell death. In addition, there were no changes in Bax levels (whole cell lysates) after treatment with both sesquiterpene lactones (Figure 4a), but there was a decrease in Bax levels in the cytosolic fraction after treatment with 30 µM sesquiterpene lactone **2** for 24 h. This was associated with the increase of Bax in mitochondria-enriched fraction. This Bcl-2 family protein is characterized by its ability to form pores across the outer mitochondrial membrane [16]. An interesting result of these experiments was that both sesquiterpene lactones downregulate the Bcl-2 levels (Figure 4a) and this could be the mechanism by which these germacranolides block the protection conferred by Bcl-2. This anti-apoptotic protein plays a crucial role in apoptosis induction by antagonizing the mitochondrial outer membrane permeability. In addition, the inhibition of Bcl-2 in acute myeloid leukemia may overcome chemoresistance without affecting normal hematopoietic stem cells.

Activation of caspase-8 may result in the proteolytic cleavage of Bid, a Bcl-2 family protein, which translocates to mitochondria to release cytochrome *c* [24]. A significant decrease in full-length Bid after treatment was observed for both sesquiterpene lactones, in accordance with the processing and activation of caspase-8.

To determine whether cell death was associated with significant changes in mitochondrial membrane potential (ΔΨm), U-937 cells were treated with these compounds for 4 h or 24 h and analyzed by flow cytometry after staining with JC-1. The results indicated an important loss of ΔΨm at 4 h of treatment with compound **2** (Figure 4c), suggesting that the alteration of ΔΨm contributed to apoptosis induced by this sesquiterpene lactone. In these experiments, the protonophore carbonyl cyanide *m*-chlorophenylhydrazone (CCCP) was used as a positive control.

### 2.4. Spiciformin (**1**) and Spiciformin Acetate (**2**) Activate Mitogen-Activated Protein Kinases (MAPKs) and Induce Reactive Oxygen Species (ROS) Generation

**Figure 5:**
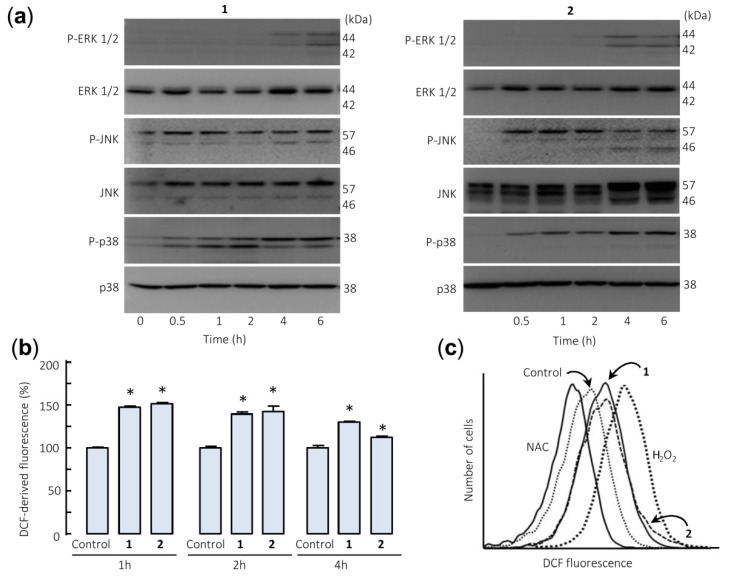
Sesquiterpene lactones induce phosphorylation of mitogen-activated protein kinases (MAPKs) and reactive oxygen species generation in U-937 cells. (**a**) Representative immunoblots show the kinetics of phosphorylation of MAPKs by the sesquiterpene lactones. Cells were incubated with the specified compound (30 µM) for the indicated times and protein extracts were prepared and analyzed by immunoblotting with specific antibodies. Blots were subsequently stripped and reprobed for the expression of total MAPKs, as indicated, to ensure equivalent loading and transfer of protein. (**b**) Cells were treated with compounds **1** or **2** (30 µM) for the indicated times (**b**) or for 1 h (**c**) and the fluorescence obtained by the oxidation of 2′,7′-dichlorodihydrofluorescein diacetate (H_2_-DCF-DA) was determined by flow cytometry. The data in (**b**) represent means ± SE of three independent experiments, each performed in triplicate (* *p* < 0.05, significantly different from untreated control). The free radical scavenger *N*-acetyl-l-cysteine (NAC, 5 mM) and H_2_O_2_ (200 µM) were used as negative and positive controls of reactive oxygen species, respectively.

The effects of sesquiterpene lactones **1** and **2** on the activation of the mitogen-activated protein kinases (MAPKs) were investigated because these enzymes play a crucial role in survival and cell death. To this end, U-937 cells were treated for different time periods and phosphorylation of the three major protein kinases of this signal transduction pathway were determined by Western blot. In an attempt to identify the primary targets and early mechanism of action of sesquiterpene lactones, we interrogated the MAPK pathway activation by using high concentrations of the drugs (> antiproliferative IC_50_ values). The results showed that sesquiterpene lactones induce fast phosphorylation (0.5 h) of JNK/SAPK (c-Jun *N*-terminal kinases/stress-activated protein kinases) and p38^MAPK^ (p38 MAPKs) and the activation of ERK (extracellular signal-regulated kinases) 1/2 took place after 4–6 h under identical experimental conditions (Figure 5a). These results indicate that both sesquiterpene lactones induce activation of the three main mitogen-activated protein kinases following different kinetics. Fast activation of JNK/SAPK and p38^MAPK^ has been reported to trigger cell death in response to several cellular stressors including oxidative stress [25,26].

The involvement of reactive oxygen species (ROS) in the apoptosis induced by germacranolides **1** and **2** was investigated because they are considered important mediators and are generated by many apoptosis-inducing agents. To this end, U-937 cells treated with both sesquiterpene lactones were stained with 2′,7′-dichlorodihydrofluorescein diacetate (H_2_-DCF-DA) and analyzed by flow cytometry. The results revealed a fast increase (1 h) in ROS levels in sesquiterpene lactones-treated cells (Figure 5b,c). In these experiments, the antioxidant *N*-acetyl-l-cysteine (NAC, 5 mM) and hydrogen peroxide (200 µM) were used as negative and positive controls. 

## 3. Materials and Methods

### 3.1. Sesquiterpene Lactones Evaluated

The extraction and isolation of spiciformin (**1**) from the aerial parts of *Tanacetum ptarmiciflorum* and *Tanacetum ferulaceum* var. *latipinnum* was performed as previously described [20]. The acetyl derivative of spiciformin (**2**) was obtained by treatment with acetic anhydride and pyridine and purified by column chromatography over silica gel using *n*-hexane-ethyl acetate as eluent. Known compounds spiciformin and spiciformin acetate were identified by HR-MS (high resolution mass spectrometry) as well as ^1^H- and ^13^C-NMR spectrometry. The previously unreported ^13^C-NMR data of spiciformin acetate were obtained on a Bruker model AMX-500 spectrometer with standard pulse sequences operating at 126 MHz. ^13^C-NMR (126 MHz, CDCl_3_) δ = 127.8 (C-1), 23.5 (C-2), 35.2 (C-3), 62.3 (C-4), 55.6 (C-5), 75.6 (C-6), 57.3 (C-7), 76.3 (C-8), 34.8 (C-9), 127.5 (C-10), 134.3 (C-11), 124.3 (C-12), 167.3 (C-13), 15.9 (C-14), 16.5 (C-15), 173.1 (CH_3_-C=O), 22.4 (CH_3_-C=O).

### 3.2. Cell Culture and Cytotoxicity Assays

The U-937 (ACC 5) and HL-60 (ACC 3) human acute myeloid leukemia and SK-MEL-1 (ACC 303) human melanoma cells were obtained from the German Collection of Microorganisms and Cell Cultures (Braunschweig, Germany) and cultured in RPMI-1640 medium containing 2 mM l -glutamine supplemented with 10% heat-inactivated fetal bovine serum and 100 µg/mL streptomycin and 100 U/mL penicillin in 5% CO_2_ at 37 °C [27,28]. The U-937 cell line overexpressing human Bcl-2 (kindly provided by Dr. Jacqueline Bréard, INSERM U749, Faculté de Pharmacie Paris-Sud., Châtenay-Malabry, France) was cultured as described [28]. The functioning of U-937/Bcl-2 cells was checked with the cytotoxic agent 1-β-d-arabino furanosylcytosine (1 µM, 24 h) as previously described [29]. Mononuclear cells from peripheral blood were isolated from heparin-anticoagulated blood of healthy donors by centrifugation using a Ficoll-Paque Plus density gradient (GE Healthcare Bio-Sciences AB, Uppsala, Sweden). All donors gave informed consent (CEIC-CHUIMI-2015/780, 13 August 2015. Ethics committee of clinical research of the C.H.U. Insular-Materno Infantil of Gran Canaria). The Vero cell line (ATCC, CCL81) was cultured in Dulbecco’s modified Eagle’s medium containing 10% (v/v) fetal bovine serum, 2 mM l-glutamine, 100 U/mL penicillin, and 100 µg/mL streptomycin.

The cytotoxicity of sesquiterpene lactones on human tumor, human mononuclear cells, and Vero cells was determined by colorimetric 3-(4,5-dimethyl-2-thiazolyl)-2,5-diphenyl-2*H*-tetrazolium bromide (MTT) assay [30]. Cells (5000) were seeded in 96-well microculture plates with increasing concentrations of germacranolides for 24 or 72 h at 37 °C. The IC_50_ values were determined graphically for each experiment as described previously [27]. Values are means ± standard errors of the means S.E.M. from at least three independent experiments, with three determinations each.

### 3.3. Evaluation of Apoptosis

Fluorescent microscopy, flow cytometric analysis of annexin V-FITC and propidium iodide-stained nuclei were performed as described [28]. Briefly, for fluorescent microscopy, cells were washed with phosphate buffered saline PBS, fixed in 3% paraformaldehyde, and stained with 20 µg/mL Hoechst 33258. For flow cytometry of propidium iodide-stained cells, cells were fixed in 70% ethanol overnight at −20 °C and then washed in PBS, incubated in the dark with 1 U/mL RNase A (DNase free) and 10 µg/mL of propidium iodide at room temperature. Double staining with annexin V-fluoresceine isothiocyanate (FITC) and propidium iodide was performed using an Annexin V-FITC apoptosis detection kit (BD PharMingen, San Diego, CA, USA) according to the manufacturer’s protocol. All flow cytometry assays were performed using a BD FACSVerse cytometer (BD Biosciences, San Jose, CA, USA).

### 3.4. Western Blot Analysis and Subcellular Fractionation

Western blot analyses of caspases, PARP, Bcl-2 family members, and MAPKs and cytochrome *c* release from mitochondria were performed as described previously [28]. Briefly, for whole-cell lysates cells were resuspended in lysis buffer (20 mM Tris-HCl (pH 7.4) containing 1% Triton X-100, 20 mM sodium β-glycerophosphate, 10 mM NaF, 2 mM EDTA, 2 mM tetrasodium pyrophosphate, 10% glycerol, 137 mM NaCl, 2 mM sodium orthovanadate, and protease inhibitors (1 mM phenylmethylsulfonyl fluoride and 1 µg/mL leupeptin and aprotinin)), sonicated, and centrifuged. For the subcellular fractionation to determine cytochrome *c* release, cells were resuspended in ice-cold buffer [20 mM HEPES (pH 7.5), 250 mM sucrose, 10 mM KCl, 1.5 mM MgCl_2_, 1 mM EGTA, 1 mM EDTA, 1 mM dithiothreitol, 0.1 mM phenylmethylsulfonyl fluoride, and 1 µg/mL aprotinin, leupeptin, and pepstatin A], lysed by being pushed them several times through a 22-gauge needle, and the lysate was centrifuged at 1000× *g* for 5 min at 4 °C to eliminate nuclei and unbroken cells. The resulting supernatant was centrifuged to 10,000× *g* at 4 °C for 20 min to obtain the mitochondrial fraction. The supernatant was centrifuged again at 105,000× *g* for 45 min at 4 °C and the resulting supernatant was used as the soluble cytosolic fraction. 

Samples containing equal amounts of proteins were separated by electrophoresis, transferred to poly(vinylidene difluoride) membranes, and probed with specific antibodies. The primary antibodies used for Western blots were purchased from the following companies: anti-caspase-3 (ADI-AAP-113), -7 (ADI-AAM-137), -8 (ADI-AAM-118), and -9 (ADI-AAM-139) from Enzo (Plymouth Meeting, PA, USA); anti-caspase-4 monoclonal antibody (M029-3) from Medical and Biological Laboratories (Nagoya, Japan); anti-poly(ADP-ribose) polymerase (PARP) (551024) and anti-cytochrome *c* (556433) from BD Pharmingen (San Diego, CA, USA); anti-Bcl-2 (#2872), anti-Bax (#2772), anti-Bid (#2002), anti-JNK/SAPK (#9252), antiphospho-JNK/SAPK (phosphor T183 + Y185) (#9251), anti-ERK1/2 (#9102), antiphospho-ERK1/2 (T202/Y204) (#9101), anti-p38^MAPK^ (#9212), and anti-phospho-p38^MAPK^ (T180/Y182) (#9211) antibodies from Cell Signaling Technology (Beverly, MA, USA); anti-β-actin (clone AC-74, A2228) and anti-α-tubulin (clone B-5-1-2, T6074) from Sigma-Aldrich (Saint Louis, MO, USA); mouse monoclonal COX IV antibody (mAbcam33985) from Abcam (Cambridge, UK). Secondary antibodies (NA9310 and NA9340) were from GE Healthcare (Little Chalfont, UK). Polyvinylidene difluoride (PVDF) membranes were from Millipore (Temecula, CA, USA).

### 3.5. Assays of Caspase Activity

Caspase-3/7, caspase-8, and caspase-9 activities were determined by measuring proteolytic cleavage of specific colorimetric substrates. Specifically labeled substrates for caspase-3 like protease, -8 and -9 activities were DEVD-*p*NA (*N*-acetyl-Asp-Glu-Val-Asp-*p*-nitroaniline), IETD-*p*NA (*N*-acetyl-Ile-Glu-Thr-Asp-*p*-nitroaniline), and LEHD-*p*NA (*N*-acetyl-Leu-Glu-His-Asp- *p*-nitroaniline), respectively. Equal amounts of protein were used and absorbance was recorded at 405 nm as previously described [28].

### 3.6. Analysis of Mitochondrial Transmembrane Potential (Δψ_m_) and Determination of Intracellular Reactive Oxygen Species (ROS) Levels

The mitochondrial membrane potential and ROS levels were determined by flow cytometry using the fluorochromes JC-1 (5,5′,6,6′-tetrachloro-1,1′,3,3′-tetraethylbenzimidazolylcarbocyanine iodide) and H_2_-DCF-DA (2′,7′-dichlorodihydrofluorescein diacetate), respectively. Fluorescence of JC-1 was detected at excitation and emission wavelengths of 527 and 590 nm, respectively. Fluorescence of 2′,7′-dichlorofluorescein was detected at excitation and emission wavelengths of 488 and 530 nm, respectively. Internal controls using unlabeled cells indicated that spiciformin (**1**) and spiciformin acetate (**2**) autofluorescence was null at all assayed conditions. All of the methods have been described in detail elsewhere [28].

### 3.7. Statistical Analysis

Statistical differences between means of control and treated samples were tested using Student’s *t*-test (two samples) or one-way ANOVA (3 or more samples) with Tukey’s test used for *a posteriori* pairwise comparisons of means. *P*-values below 0.05 were considered as statistically significant.

## 4. Conclusions

In conclusion, spiciformin (**1**) and spiciformin acetate (**2**) are cytotoxic against the human acute myeloid leukemia cells, including cells that overexpress Bcl-2, and display less cytotoxicity against the melanoma cell line SK-MEL-1 and mononuclear cells isolated from healthy volunteers. Human U-937 cells responding to germacranolides **1** and **2** manifested a great reduction in the levels of the anti-apoptotic protein Bcl-2. The mechanism of cytotoxicity triggered by these sesquiterpene lactones was due to caspase-dependent apoptosis and associated with mitogen-activated protein kinase pathway activation and reactive oxygen species generation. The results support that these sesquiterpene lactones have an impact in at least two cellular hallmarks of cancer, such as resistance to apoptosis and sustained proliferative signaling. However, the early mode of action of these compounds and relative signaling cascade remains to be elucidated in more detail.

## Figures and Tables

**Table 1:** Effects of sesquiterpene lactones on cell viability of human tumor cell lines.

	IC_50_ (µM)			
	U-937	HL-60	SK-MEL-1	U-937/Bcl-2
Spiciformin (**1**)	5.0 ± 0.8	4.7 ± 1.3	22.5 ± 4.1	5.5 ± 1.4
Spiciformin acetate (**2**)	1.2 ± 0.5	3.8 ± 1.5	11.5 ± 4.0	1.6 ± 1.0

U-937, U-937/Bcl-2, HL-60: acute myeloid leukemia; SK-MEL-1: melanoma. The IC_50_ values were calculated using the methodology described in the Materials and Methods section from cells treated for 72 h. The data shown represent the mean ± SEM (standard error of the mean) of 3–5 independent experiments with three determinations each.
